# *Syntrichia caninervis* adapt to mercury stress by altering submicrostructure and physiological properties in the Gurbantünggüt Desert

**DOI:** 10.1038/s41598-022-15822-2

**Published:** 2022-07-09

**Authors:** Yuqing Mao, Weiguo Liu, Xiaodong Yang, Yaobao Chang, Tao Yang, Xiyuan Wang, Yinguang Chen

**Affiliations:** 1 College of Ecology and Environment, Xinjiang University, Ürümqi, 830017 China; 2 Key Laboratory of Oasis Ecology of Education Ministry, Ürümqi, 830017 China; 3 Xinjiang Jinghe Observation and Research Station of Temperate Desert Ecosystem, Ministry of Education, Ürümqi, 830017 China; 4 Department of Geography and Spatial Information Technology, Ningbo University, Ningbo, 315211 China; 5 School of Environment Science and Engineering, Tongji University, Shanghai, 200092 China

**Keywords:** Ecophysiology, Plant physiology, Plant stress responses

## Abstract

Sewage and industrial waste discharges have been found to have a deleterious effect on plant growth and environmental safety through the accumulation of trace metal mercury (Hg) in soils. Although the effects of Hg on vascular plants have been reported in terms of enzyme activity, oxidative damage and physiology, few studies have been done on non-vascular plants. A simulation experiment including 7 Hg concentrations (0, 10, 20, 30, 40, 50, 75 µM) was conducted to investigate the influence of Hg stress on ultrastructure and physiological properties of biocrust moss *Syntrichia. caninervis* across 7 consecutive days*.* The results showed that the lowest lethal concentration of *S. caninervis* was 30 µM Hg. The mortality rate of the plants increased significantly with Hg concentrations. The ultrastructure did not change significantly at Hg concentration ≤ 20 µM, while exceeding which, cell walls began to separate, nuclei began to blur, and chloroplasts began to expand. The soluble sugars (SS), peroxidase (POD), and superoxide dismutase (SOD) activities increased initially and then decreased with the increase of concentration in the time gradient, with the largest values at 20 µM. The contents of malondialdehyde (MDA) and proline (Pro) increased with the increase of Hg concentration, both reached peak value at 50 µM. However, chlorophyll (Chl) contents continued to decrease along both the concentration and time gradients. Pearson correlation and principal component analysis showed that two principal components (PC1 and PC2) explained 73.9% of the variance in plant adaptation to Hg stress. SOD, POD, Chl, SS, and Pro all responded well to Hg in *S. caninervis*. Our study showed that Hg stress caused changes in ultrastructure and physiological metabolism of *S. caninervis*. 20 µM was the maximum concentration of Hg that biocrust moss *S. caninervis* can tolerate. *S. caninervis* mainly adopted two adaptation strategies related to exclusion and accumulation to reduce Hg stress.

## Introduction

Plants are adversely affected by various environmental factors that impede their growth and development. Trace metals stress is a typical abiotic stress. However, the response varies for different patterns of plants and the concentration is a major factor. Due to the expending of modern industries, increasing amounts of trace metals were released into environment. For examples, from mining, chemical, and metal processing industries in the past few decades resulting in elevated concentrations of trace metals in the soil^1^. Most of the trace metals pollution are severe, long-term, and non-reversible in nature^2^. In a broad sense, trace metals are defined as metals with an atomic density of more than 5 g·cm^−3^. Among all elements discovered so far 53 elements have been classified as trance metals^3^. At high concentrations, all trace metals are toxic, but some of them, such as copper (Cu) and zinc (Zn) are essential nutrients at low concentrations. However, most of them have no beneficial function, including chromium (Cr), cadmium (Cd), lead (Pb) and mercury (Hg) that even in small quantities can exert deleterious impacts on plant metabolism^4^. Trace metals restrict plant growth by lowering the performance of different cell components, such as the thylakoid membranes of chloroplast, lipids and proteins^5^.

When the plant is subjected to trace metals stress, its cells are stimulated to produce a series of reactions that respond to stress to maintain their normal physiological activities^6^. These responses are manifested by changes in related physiological indicators, which characterize the extent to the cells are poisoned or damaged, the ability of the cells to resist and mitigate the toxicity^7^. Mercury (Hg) is considered to be one of the most dangerous trace metal elements causing serious environmental pollution and raising a carcinogenic risk to human^8^. It also poses a significant threat to ecosystem function due to their bioaccummulative, persistent and long-distance migration ability^9^. Hg negatively affect many physiological processes, including the inhibition to plant growth, the degradation of photosynthesis system, and the changes in membrane permeability, cell soluble protein, antioxidant system and leaf chlorosis^10^. Hg damage to the organelle structure and membrane permeability when its ions enter the plant cells^11^. It also changes the activity of antioxidant enzymes and antioxidants in plants due to the accumulation of reactive oxygen and lipid peroxidation caused by toxicity^12^. To minimize the damage and toxicity, plants have developed some mechanisms to prevent oxidative damage caused by Hg pollution. For example, plants can change lipid composition, antioxidant enzyme activity, sugar or amino acid content, and improve the quantity of soluble proteins to decrease the toxicity of Hg^13^. At present, many studies have reported the effects of Hg on enzyme activity, oxidative damage and physiology of plants from different aspects^14,15^, but these studies mainly focus on vascular plants, while regarding non-vascular plants its potential effects are relatively poorly understood.

Biological soil crusts (BSCs) are a special group of non-vascular plants, which are composed of a group of organisms dominated by cyanobacteria, green algae, lichens and mosses^16^. BSCs usually grow in arid and semi-arid desert ecosystem, and played an important role in the maintenance and stability of ecosystem functions^17^. The presence of BSCs are beneficials to soil stability, nutrient availability, biogeochemical cycles, seedling establishment, food and habitats supply for reptiles and invertebrates^18^. Among all types of BSCs, mosses crusts are the most common in the world, *Syntrichia caninervis* is widely distributed across the drylands of the Northern Hemisphere from the Mojave Desert in the United the States through parts of Europe, to the Gurbantünggüt Desert in China^19^. Till now, there are many studies on tolerance strategy to drought^20,21^, and the response of physicochemical properties and spectrally monitoring on environmental change^22,23^. However, few studies have looked at the tolerance mechanisms of *S. caninervis* to Hg toxicity.

The aim of this study is to present a better understanding of the physiological responses of *S. caninervis* BSCs to the Hg toxicity. In order to accomplish it, we analyzed the ultrastructure of *S. caninervis* after exposed to Hg at different concentrations for 7 days. We also examined the responses of 7 physiological properties (i.e., proline accumulation, soluble sugar, chlorophyll, cell ultrastructure, malonydialdehyde, peroxidase and superoxide dismutase activities) of *S. caninervis* on the Hg addition. The results from this study will help reveal the response mechanisms of *S. caninervis* to Hg toxicity, having an important theoretical significance for the protection and management of *S. caninervis* in desert ecosystem.

## Materials and methods

### Study site

This study was conducted in the southern margin of the Gurbantünggüt Desert (44°21′–46°10′ N, 84°00′–90°31′ E), which is located in the center of the Junggar Basin, Xinjiang Uygur Autonomous Region, Northwest China. The study site with a typical temperate continental climate with a mean annual temperature ranging from 5 to 10 °C, and the mean annual precipitation range from 100 to 150 mm. The mean annual potential evaporation reaches 2000 mm, while the relative humidity ranges from 40 to 60%^23^. The most common vascular plants included *Haloxylon ammodendron*, *Erodium oxyrrhynchum*, *Salsola ruthenica* and *Calligonum leucocladum*; which are the builders of native vegetation. The soil surface of this area is mainly covered by well-developed BSCs. Moss crusts and *S. caninervis* are dominant community and species of BSCs in the study site, respectively^24^.

### Hg concentrations

The concentration of Hg used in previous studies related to negative effects on plants ranged up to 100 µM^25–27^. Therefore, we conducted a pre-experiment with Hg concentration gradients (0, 5, 10, 15, 20, 25, 30, 35, 40, 45, 50, 55, 60, 65, 70, 75, 80 µM, the Hg treatment, obtained using HgCl_2_ (Analytical Reagent) water (DI) solution). Results showed that Hg concentrations below 75 µM did not immediately lead to the death of *S. caninervis*, however all the individuals died on the 7th day of 75 µM Hg. Thus, the maximum concentration was selected at 75 µM Hg. Seven Hg treatments were conducted in our experiment: six Hg treatments (10, 20, 30, 40, 50, 75 µM) and a control (CK, 0 µM).

### Plant culture and treatment

*S. caninervis* were sampled in May 2019 by using a small shovel at the southern margin of the Gurbantünggüt Desert. More specifically, the *S. caninervis* with good growth and relatively uniform were firstly selected as sampling objects. Then, the surface sand was gently removed with a brush, and the samples were collected in petri dishes. After that, they were taken to the laboratory and cultured with deionized water for 2 days in an incubator at 25 ± 1 °C and 20% relative humidity (RH)^28^*.* After culturing for 2 days, *S. caninervis* individuals with about 0.8 cm in height were selected for further experiment using forceps, all samples (each sample includes 75 individuals) were placed in numbered petri dishes (d = 15 cm, V = 512.21 cm^3^). They were treated with 0, 10, 20, 30, 40, 50, 75 µM Hg obtained from HgCl_2_ water (DI) solution, and all roots should be immersed in solution. Finally, all samples were placed back to the incubator. All samples were cultured for 7 days, and the growth conditions of *S. caninervis* were observed on every day. During the experimental period (7 days), HgCl_2_ solutions with each respective concentration were added into the petri dishes each day in order to maintain the Hg stress. Each Hg addition treatment was replicated three times with a total of 21 samples.

### Determination of Hg contents

*S. caninervi* with different treatments of Hg after 7 days were collected and washed. Subsequently, they were air dried and grounded into powder with a mortar and pestle, 0.2 g dry samples were was taken in digestion tubes and 10 mL of HNO_3_, HClO_4_ and H_2_SO_4_ (5:1:1) were added to each tube and were kept for 12 h. The tubes were then placed at 80 °C in the digestion block for about 1 h and then raised the temperature slowly to 120–130 °C. When digestion was completed, the solutions were cooled, filtered and diluted to 100 mL with double deionized water. In the filtrates, the Hg was assayed using an atomic absorption spectrometer (Analyst 700 of Perkin Elmer)^29^.

### Transmission electron microscopy (TEM)

According to Khan et al.^30^, samples were prepared for the investigation of changes in ultrastructure along the concentration gradient of Hg addition. Briefly, the top freshest sections (at least > 2 mm^2^) of *S. caninervis* were randomly cut out from *S. caninervis* individual. Then, the sections were fixed with 1 mL of 2.5% glutaraldehyde in PBS 0.1 M phosphate buffer (pH 7.4), and stored at 4 °C. After that, the sections were rinsed several times using phosphate buffer solution (PBS), and were fixed in 1% (v/w) OsO4 for 1.5 h. Then, PBS 0.1 M (pH 7.4) were used again to wash several times with intervals. After that, the sections were dehydrated using a graded ethanol series (30, 50, 60, 70, 80, 90, 95, and 100%) with 15–20 min intermittence. The sections were placed in an epoxy mixture for 1 h and embedded in fresh epoxy resin capsules and polymerized at 60 °C overnight. After resin embedding, ultrathin sections (80–100 nm) were cut and stained on uranyl acetate and lead citrate, and collected on 200 mesh copper grids. The sections were observed with a TEM (JEM-1200EX) operating at 80 kV.

### Physiological and biochemical measurements

In the experimental period of this study, the physiological properties were measured daily for each sample under each treatment. In order to reduce the influence of stress time on the measurement results, all samples used for the measurement of physiological properties were collected at the same time each day. The measurement of each property under each experimental treatment was repeated 3 times. The test methods of physiological properties are introduced as follows.

#### Determination of chlorophyll contents

Chlorophyll *a* and *b* contents were determined by the method of Arnon^31^. Briefly, samples (0.3 g) were homogenized in acetone under dark conditions. Homogenates were centrifuged at 2800 *g* for 5 min, and the absorbance of the supernatant was measured at 645 and 663 nm. Then, the chlorophyll *a* and *b* contents and the total chlorophyll were determined. The total chlorophyll concentration was calculated utilizing the following formula: (12.7*A*_663_ – 2.69*A*_645_ + 22.9*A*_645_ – 4.68*A*_663_).

#### Determination of proline and soluble sugar

Proline (Pro) content was measured according to the method of Bates et al.^32^. A quantity (0.5 g) of fresh aboveground material of *S. caninervis* was homogenized with 3% sulfosalicylic acid (5 mL) on ice. The homogenate was heated in a water bath at 100 °C for 15 min and then cooled to indoor temperature. The extract was centrifuged for 10 min at 4000 *g*. The supernatant (2 mL) was mixed with the same volumes of glacial acetic acid (2 mL) and acid ninhydrin (2 mL), and then held the mixed liquids in a bath at 100 °C for 1 h. The reaction mixture was extracted with toluene (4 mL) and absorbance was measured at 520 nm using a spectrophotometer. The Pro content was calculated from the standard curve.

Soluble sugar (SS) content was measured by anthrone test^33^. Fresh samples (0.5 g) was extracted using 5 mL of distilled water in a boiling water bath for 10 min, and then centrifuged it at 4000*g* for 10 min. The reaction included 0.05 mL of supernatant, 0.95 mL of ethanol (80%, v/v), and 5 mL of anthrone (0.1%, v/v) and was incubated in a boiling water bath for 10 min. After cooling, the absorbance of solution was measured at 625 nm.

#### Determination of malondialdehyde contents

Malondialdehyde (MDA) contents were measured by thiobarbituric acid based on the colorimetric method described by Choudhury and Panda^34^. The reaction mixture consisted of 1 mL enzyme solution, 1 mL 50 mM phosphate buffers (pH 7.0) and 2 mL 10% trichloroacetic acid was heated at 100 °C for 15 min and then centrifuged at 4000*g* for 10 min. The absorbance of the supernatant was measured at 450 nm, 532 nm and 600 nm by a using spectrophotometer.

#### Assay of antioxidant enzymes

The superoxide dismutase (SOD) and peroxidase (POD) activities were measured based on the methods of Kumar et al.^35^ and Pasquariello et al.^36^, respectively. Briefly, 0.5 g fresh sample was ground in an ice-cold mortar with enzyme extraction buffers (2 mL). The mixture was later centrifuged at 12,000*g* for 20 min at 4 °C. The supernatant was used for the measurement of POD and SOD activities. The SOD activity was determined by measuring the ability of the enzyme to inhibit the photochemical reduction of nitroblue tetrazolium. The absorbance was set to 560 nm and test value was recorded using a spectrophotometer. For the measurement of POD activity, the methylcatechol reaction mixture was added to the supernatant while the absorbance was set at 470 nm.

### Statistical analysis

All experimental data were presented as the mean ± *SD*. One-way ANOVA was used to test differences in physiological properties among the different concentrations of Hg addition. Differences between two groups were measured using Duncan test. The significance of differences was assessed at the 5% probability level (*p* < 0.05). Correlation analysis and principal component analysis (PCA) were performed to test the relationship between physiological properties, and the adaptive strategies of *S. caninervis* to Hg toxicity. All data analyses were performed using SPSS 19.0 software.

### Permission statement

This is a permission from Xinjiang University to collect samples of well development *Syntrichia caninervis* from the southern margin of the Gurbantünggüt Desert, were complied with relevant institutional, nation, and international guidelines and legislation and transferring them safely to physico-chemical analysis and ultrastructure determination to Xinjiang University, College of Ecology and Environment. Mamtimin Sulayman formally identified the *Syntrichia caninervis* used in the study. The voucher specimen of the *Syntrichia caninervis* is not deposited in the public herbarium.

## Results

### Effects of different Hg stresses on the S. caninervis growth condition

The effect of Hg on *S. caninervis* growth condition was evaluated by color changes*.* With the increase in the concentrations of Hg addition, *S. caninervis* would lose their green color and then became yellowish or black-brown due to greater growth retardation. Black-brown indicated that the individuals were dead. According to the observed survival of *S. caninervis* along the experimental period (7 days) (Table 1), our results showed that the most *S. caninervis* individuals were green or yellow-green (no death was observed) while the concentrations Hg was less than 30 µM. In contrast, when Hg concentration was equal to or higher than 30 µM, the black-brown death individuals appeared after 5 days. As the increased in Hg concentration, the mortality rate of *S. caninervis* individuals increased continuously. Even on the 7th day, all plants died in 75 µM solution (Table 1).

**Table 1:** The mortality rate of *S. caninervis* in the different concentrations of Hg addition.

Hg concentration µM	Mortality rate (%)						
	Day1	Day2	Day3	Day4	Day5	Day6	Day7
0	0	0	0	0	0	0	0
10	0	0	0	0	0	0	0
20	0	0	0	0	0	0	0
30	0	0	0	0	0	4	12
40	0	0	0	0	4	36	72
50	0	0	0	0	20	44	84
75	0	0	0	0	32	88	100

### Hg accumulation

The content of Hg in tissues of *S. caninervis* increased concurrently with the increase of external Hg concentration (Table 2). After 7 days of culture with different Hg treatments, the Hg accumulation of *S. caninervis* was 1.22–20.28 µg/g dry weight. The maximum accumulation of Hg was 20.28 µg/g dry weight in *S. caninervis* treated with 75 µM Hg.

**Table 2:** The Hg accumulation of *S. caninervis* with different treatments of Hg after 7 days.

Hg concentration (µM)	Hg content (µg/g dry weight)
0	NA
10	1.23 ± 0.01a
20	2.04 ± 0.26a
30	6.81 ± 0.36b
40	11.09 ± 1.22c
50	17.03 ± 1.59d
75	18.18 ± 2.10d

Data are means with standard errors (n = 3), different lowercase letters indicate a significant difference among seven Hg treatments after 7 days (*p* < 0.05).

### Effects of different Hg stresses on the ultrastructure

**Figure 1:**
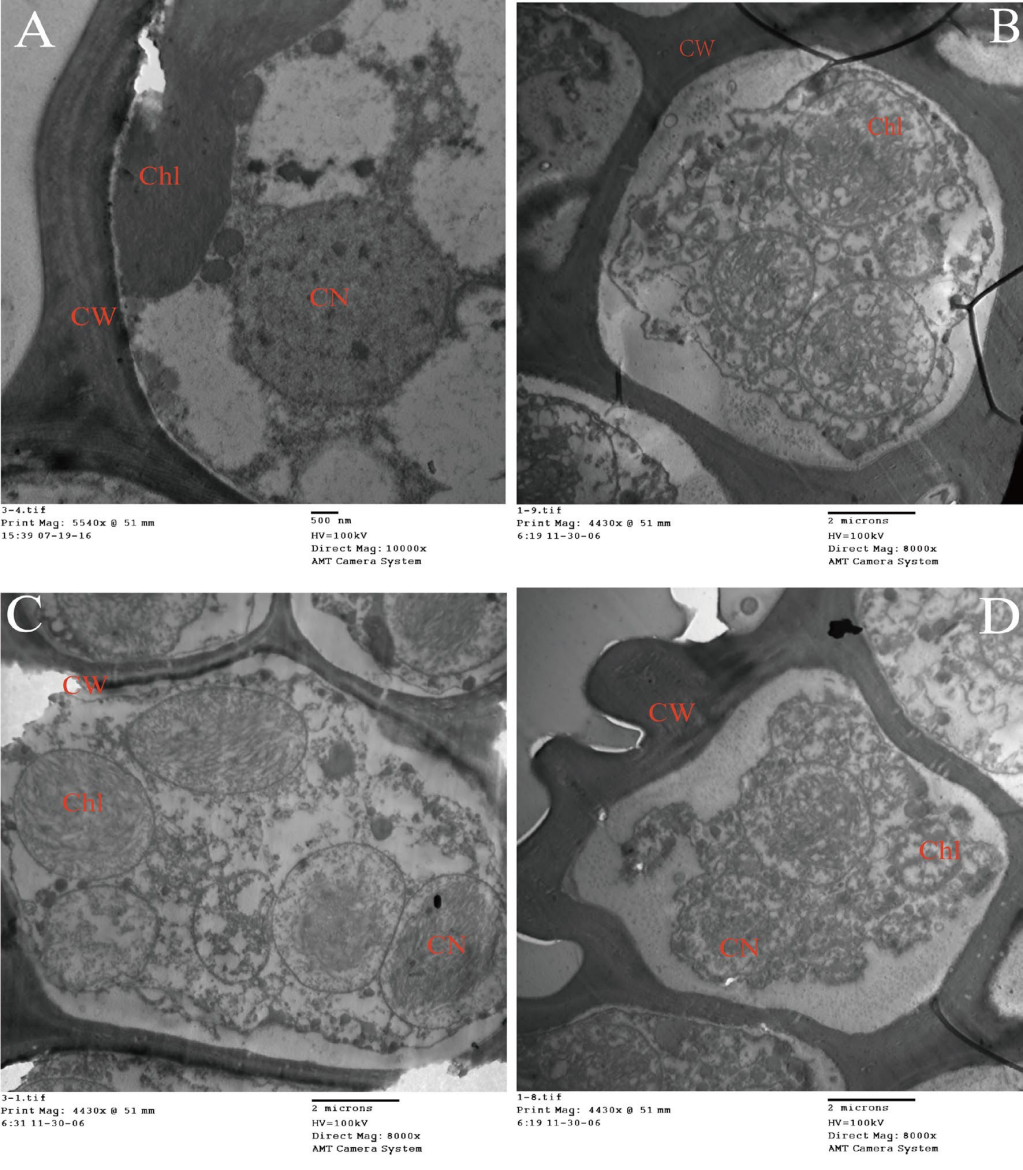
Change in cell ultrastructure in the leaves of *S. caninervis* along with Hg stress concentration by measured using transmission electron microscopy (TEM). (**A**) control; (**B**) 20 µM Hg concentration; (**C**) 30 µM Hg concentration; (**D**) 50 µM Hg concentration. *Chl* chloroplast, *CN* cell nucleus, *CW* cell wall, *CM* cell membrane, *SG* starch grain.

TEM analysis illustrated that the Hg stress changed the ultrastructure of *S. caninervis*, but the change degree depended on concentrations. The ultrastructure was compared between the control (0 µM) and low (20 µM), medium (30 µM) and high (50 µM) concentrations. More specifically, there are no structural alterations in the control group (0 µM) (Fig. 1A). The well-developed cell wall (CW), cell membrane (CM), cell nucleus and chloroplast (Chl) can be clearly seen in TEM images. The thylakoids were arranged regularly and tightly in the grana. Chloroplast has an obvious appearance and clear oval shape. Starch grains (SG) with small diameters were rarely observed in cells. In contrast, the ultrastructure changed obviously, and the cell shape became irregular in 20, 30 and 50 µM of Hg concentrations. However, the degree of ultrastructural change was also closely related to the concentration. Briefly, under the stress of 20 µM Hg concentration, chloroplast morphology was good, but the cell membrane was slightly damaged, the nucleus (CN) was slightly deformed, the numbers of starch grains were decreased, and the nucleoli was still mounted in cell (Fig. 1B). However, after increasing Hg concentration larger than 30 µM, compared with the control and 20 µM groups, the thylakoid shape was swollen, and the thylakoid lamellae were loose and arranged randomly. Also, the unstable cell wall (CW) with scattered cell membrane (CM), the invisible nucleus (CN), nucleolus and nuclear membrane (NM), as well as the absence of starch grains were observed from TEM images (Fig. 1C,D).

### Effects of different Hg stresses on the physiological properties

**Figure 2:**
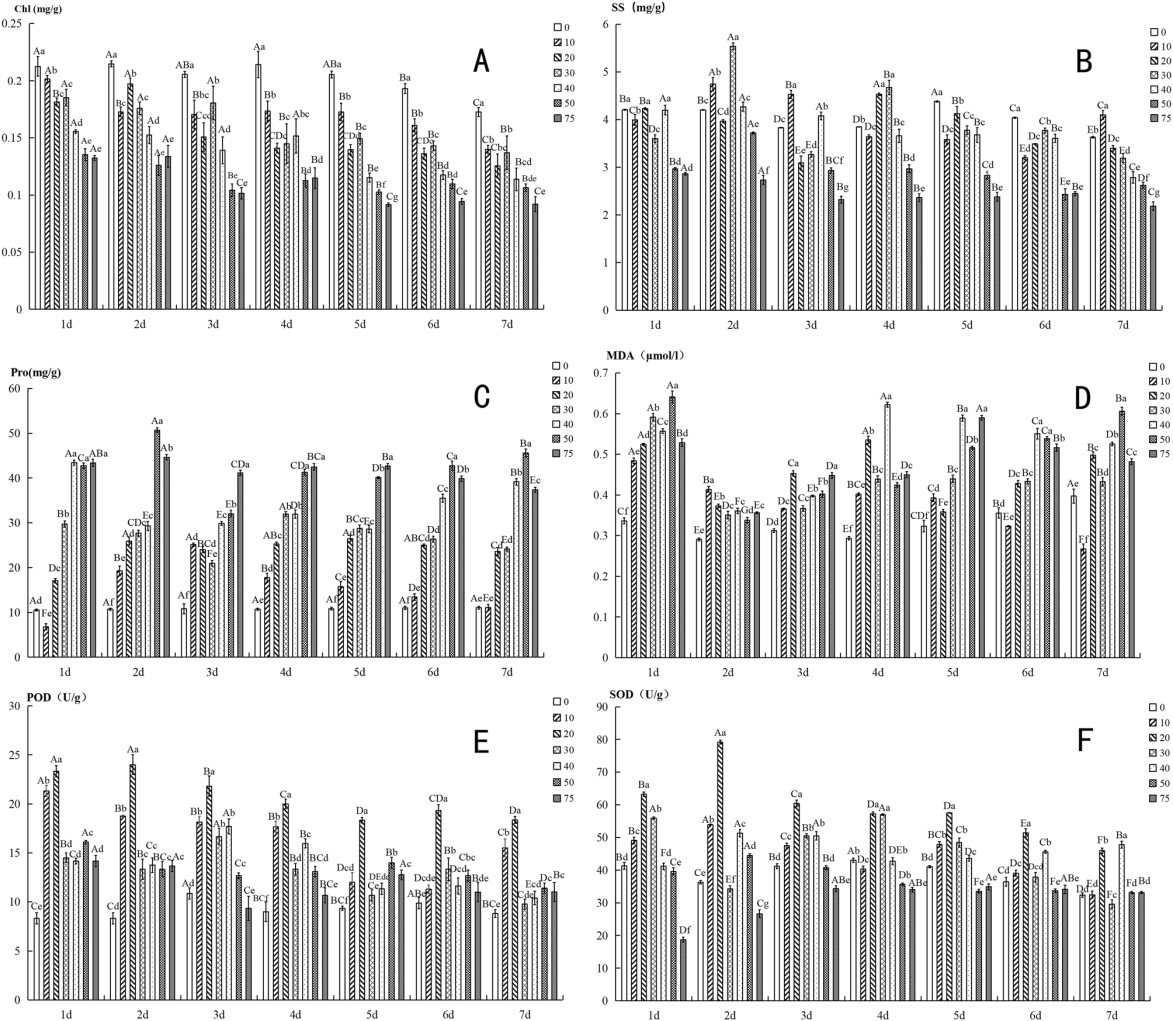
Differences in physiological properties among Hg addition concentrations. (**A**) Chlorophyll (Chl); (**B**) soluble sugar (SS); (**C**) proline (Pro); (**D**) malondialdehyde (MDA); (**E**) peroxidase (POD); (**F**) superoxide dismutase (SOD). Each value is showed using the mean of three samples (Mean ± *SD*). Different capital letters indicate a significant difference (*p* < 0.05) among seven sampling days with the same treatment; different lowercase letters indicate a significant difference (*p* < 0.05) among seven Hg treatments in the same day. Vertical bars show the standard error (SD) (n = 3).

Our results suggested that Hg stress have the significantly influences on physiological properties of *S. caninervis*. The chlorophyll (including chlorophyll *a* and chlorophyll *b*) contents decreased continuously with the increase in Hg concentration in all experimental period (from 1 to 7 days), whereas the responses of other properties were differed. More specifically, Pro content increased with Hg concentration in all experimental periods (Fig. 2C). The responses of SS, MDA, POD and SOD contents on Hg stress concentration were fluctuated (Fig. 2B,D,E,F). Specifically, the values of SS content were highest in the middle of the Hg addition concentration (30 or 40 µM), while were lower in small and large concentrations from first to third days of the experimental period. However, in the period from 4 to 6th days, the relationship between SS content and Hg addition concentration showed two completely different patterns with 30 µM as the cut-off point. SS content increased with the increase of Hg concentration when that was less than 30 µM, whereas decreased while concentration was higher than the cut-off point. On the 7th day, SS content decreased with the increase of Hg addition concentration. Besides, SS contents of 50 and 75 µM were smaller than those of the other concentrations in all experimental periods (Fig. 2B). Except for the 2th and the 4th days of the experimental period, MDA content increased continuously with the increase of Hg addition concentration. However, on the 2th and the 4th days, MDA content were the largest at 20 µM and 40 µM, while the smallest in the control (0 µM), but the intermediate in other concentrations (Fig. 2D). Similar with SS, the responses of POD and SOD contents on Hg addition concentration showed two completely different patterns in both sides of 30 µM treatment. POD and SOD contents increased with the increase of concentration below 30 µM, while showed an opposite trend when Hg addition concentrations were higher than 30 µM (Fig. 2E,F).

### Correlation analysis of physiological properties

**Figure 3:**
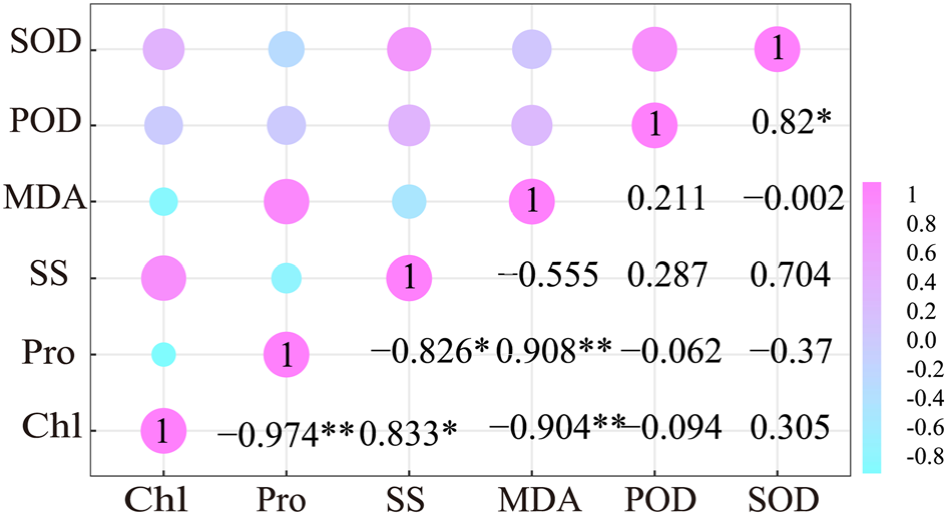
Correlation analysis among physiological properties under gradient of Hg stress. The introduction of Chl, MDA, POD, Pro, SOD and SS have showed in Fig. 3. Significance levels are denoted with *p < 0.05, **p < 0.01.

Our results found that Pro and MDA have the negative correlations to chlorophyll (*p* < 0.01). SS had the positive correlations with chlorophyll (*p* < 0.05), whereas a negative relation to Pro (*p* < 0.05). Pro showed the positive correlations to MDA (*p* < 0.01). SOD showed a positive significant correlation with POD (*p* < 0.05) (Fig. 3).

### Principal component analysis of physiological properties

**Figure 4:**
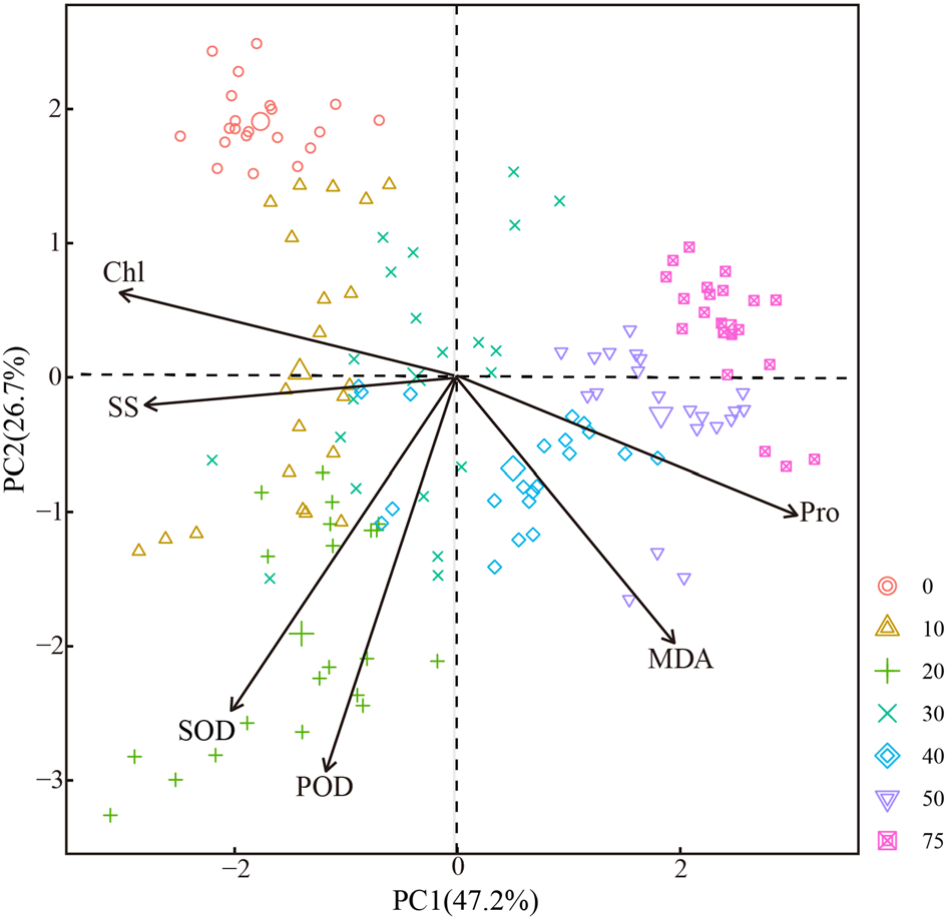
Principal component analysis of physiological properties. The introduction of Chl, MDA, POD, Pro, SOD and SS have showed in Fig. 4.

PCA scores and loading plots of the physiological properties of *S. caninervis* on the 7th day after different Hg concentrations were shown in Fig. 4. Two principal components explained 73.9% of variance in adaptation to Hg stress. The explaining variances of first and second principal components were 47.2% and 26.7%, respectively. The result showed that small distance between control (0 µM) and 10, 20 µM Hg treated samples suggest that had similar physiological properties. In contrast, larger distances between control (0 µM), 40, 50 and 75 µM Hg-treated samples demonstrates that the physiological properties in control (0 µM) were dissimilar to 40, 50 and 75 µM Hg-treated samples. At a concentration of 20 µM, SOD and POD were the main substances that were continuously adapted to Hg stress. Moreover, at a concentration of 40 µM, Pro is the most effective physiological properties adaptation to Hg stress. Meanwhile, at a concentration of 40, 50 µM, the maximum content of MDA indicates that the *S. caninervis* has been damaged to a large extent.

## Discussion

### *Response of ultrastructure* *in the mesophyll cells to Hg stress*

Many studies have showed that plant organelles would undergo a series of morphological and structural changes to adapt to the damage and stress of the trace metal elements^37–39^. For example, Sergio et al. reported that trace metal elements cause the plasmolysis and an obvious thickening of cell wall due to the harmful influences on the intracellular structure of bryophytes^40^. Vecchia et al. found that most of the damage caused by trace metal elements was related to biological membrane, resulting in that the organelles with abundance in membrane structure are more vulnerable to serious damage than other structures^25^. Among many membrane-abundance organelles, chloroplasts and mitochondria may be the most severely damaged organelles by trace metal elements, because the latter hindered the division and expansion of these two organelles, as well as affected their shape and arrangement of the cystoid system^41,42^. In this study, we have obtained the similar results to the above studies. Previous studies have demonstrated that trance metal toxicity to plants is positively correlated to trance metals concentration in plant tissues^43,44^. Our results found that Hg accumulation in *S. caninervis* increased with the addition of Hg and had the negative impacts on its growth and cell ultrastructure. The mortality rate increased continuously with the increase of Hg concentration. Under the stress and toxicity of Hg, the chloroplast, thylakoid, cell walls and cell membranes were are damaged to varying degrees, as well as the plasmolysis were occurred. Also, the damage of Hg to ultrastructure of the mesophyll cells increased with its concentration. More specifically, there were no significant change in mesophyll cells and osmiophilic granules in *S. caninervis* at Hg concentrations ≤ 20 µM. All *S. caninervis* individuals were alive and most of them showed green in color. Starch granules were the major form of energy storage. These results suggested that Hg concentrations below 20 µM were not sufficient to produce harmful influence on BSCs. After that, when the Hg concentration increased to 20 µM, intracellular starch granules disappeared, which maybe be related to the resistance of biological crust to stress and toxicity caused by trace metal elements. The decrease of starch particles reflected the increase of energy consumption, which was used to improve the tolerance of the crust to metal toxicity. Under the stress of 30 µM Hg, the mesophyll cells of *S. caninervis* gradually showed the blurred cell walls, slight division and some deformation of cytoplasm, obvious swelling and vacuolization of chloroplasts, the blurred granules and stroma lamellae, and disintegration of surrounding organelles. All these indicated that chloroplast organelles began to be damaged and became more and more serious over time. This was also reflected in the appearance of plant. *S. caninervis* showed a black-brown color, indicating they died on days 6 of the experiment. More seriously, when Hg concentration was larger than 50 µM, the concentration of Hg on the cell wall exceeded the threshold that the cells can endure, leading to the serious vacuolation formation, even chloroplast disintegration, and the fuzzy and loss of cell nuclear membrane structure, and finally resulting in cytoplasm overflow and loss of thylakoid, stroma and lamellar structure. This finding was consistent with previous studies on changes in the ultrastructure (such as cell wall, nucleus and chloroplast) regarding the stress and toxicity of trace metal elements^45^.

In this study, we found that the number of osmiophilic granules in mesophyll cells increased first and then decreased with the increase of Hg concentration. This may be because the increase of osmiophilic granules are beneficials to providing lipid for membrane renewal and further maintaining the structural integrity of the membrane at low stress of trace metal element or in the first half of our experimental period. At the same time, osmophilic granules were degraded into sugars under the environmental stress, which in turn increased the sugar concentration in cells and improved the resistance of *S. caninervis*. However, the excessive concentrations of Hg that may surpass the tolerance limit of *S. caninervis*, would result in individual death. The shutoff or the decrease of *S. caninervis* metabolism caused the reduction of the number of osmophilic granules. Additionally, our results also showed that Hg stress significantly reduced chlorophyll content (chlorophyll *a* and *b*, as well as total chlorophyll content) (Fig. 2A). This may be due to the fact that Hg stress caused the disintegration of chloroplast thylakoid, and as a result inhibited chlorophyll synthesis^46,47^.

### Response of physiological properties on Hg stress

Soluble sugar and free proline are key osmotic adjustment substances and a major indicator for plant tolerance to stress^48^. The changes of osmotic adjustment substances were conducive to maintaining the stability of cellular structure and cell osmotic pressure, particularly under severe or prolonged stress^49^. In this study, SS content increased firstly and then decreased with the increase of Hg stress. Low Hg concentration (≤ 30 µM) increased SS content, while high Hg concentration reduced it. This was probably because the increase of SS content can effectively reduce the water potential of cells and prevent dehydration caused by Hg stress in the early stage of experiment or under low concentration. In addition, starch granules can be converted into SS under the metal stress, which then increased the SS concentration in cell solution, thus enhancing the plant tolerance to metal stress. However, the exorbitant Hg stress (≥ 50 µM) may inhibit anabolism and growth of *S. caninervis*, then leading to the decrease of SS content. In addition to osmotic adjustment, Pro can also serve as molecular chaperone to stabilize protein structure and play a regulatory role in antioxidant system^50,51^. Under the condition of high metals stress, higher Pro content can maintain appropriate cell expansion pressure and protect cell structure from trace metal element damage.

In a normal growth environment, the production and removal of reactive oxygen species (ROS) are dynamic balance in plants. However, ROS balance would be disrupted if plants are stressed by drought, salinity, cold or trace metal elements. The overproduction of ROS will accumulate in plants, resulting in oxidative damage^52,53^. Antioxidant enzymes, such as POD and SOD can convert toxic superoxide radicals into harmless ions and also remove hydrogen peroxide in plants^54^. Therefore, it is considered to be an important substance for limiting ROS and improving plant tolerance to environmental stress. In our results, SOD and POD activities increased firstly and then decreased with the increase of the Hg addition concentration (Fig. 3E,F). This may indicate that the activation of ROS scavenging system was activated at the early stage of Hg stress. *S. caninervis* increased SOD and POD activities for reducing the damage of the superfluous ROS caused by the Hg stress, and enhancing the body's tolerance to Hg stress. However, the antioxidant enzyme system would be inhibited gradually with the increase of Hg stress, leading to the limitation to the scavenging ability of ROS, and then as a result decreasing SOD and POD activities due to the damages of the functional membrane and enzyme system of cells^55^. Meanwhile, as the increase of Hg stress, oxidative stress would be deteriorated and the excessive ROS could not be dissipated timely, resulting in the damage to the cytoplasmic membrane and the disruption of antioxidant enzyme system.

As an important marker of lipid peroxidation, MDA content would be increased with the reduction of the oxidative cellular defense^56^. The increase of MDA is beneficial to reduce the damage of the accumulation of toxic ROS such as OH^−^ and H_2_O_2_ caused by environmental stress to lipids, proteins and nucleic acids, thus rapid the death of plants^57^. In this study, MDA content increased with the increase of Hg stress, and the change range was higher in the later stage of the experiment than in the earlier stage. These indicated that Hg concentration has a higher induction effect on the increase of MDA content. The slow increase of MDA content in 2–4 days may be due to the fact that the activation of resistance mechanisms in plants to delay ROS damage to cells. After 5–6 days, the long-term Hg stress intensified membrane lipid peroxidation due to the inhibition of plant defense enzyme system, resulting in the rapid increase of MDA content.

### The adaptive strategies of S. caninervis to Hg stress

Plants are continuously faced with abiotic stress and have evolved various mechanisms or adaptive strategies to overcome them^58^. In this study, two main adaptive strategies of *S. caninervis* were found based on principal component analysis and Pearson correlation analyses to resist Hg stress. The first strategy is that by regulating osmotic pressure and photosynthesis. Chl and SS showed an overall decreasing trend with increasing Hg concentration. The decrease in Chl content indicates that *S. caninervis* has been subjected to the toxic effects of Hg, which inhibited photosynthesis. The increasing of SS content at Hg concentration < 30 µM improves the cytosol concentration, reduces the cellular water potential and enhances the cell water retention capacity^59^; The significant decrease in SS content under high Hg stress (> 30 µM) indicates that Hg disrupts the photosynthetic system of *S. caninervis*, causing the breakdown of soluble sugars and inhibiting their transformation. Proline plays an important role in the protection of cell membrane structures, the stability of biomolecular structures and the decreasing of cellular water potential^60,61^. In our study, the sustained increase in Pro content suggests that *S. caninervis* may reduce cellular water potential, enhance cellular water retention capacity and stabilize biomolecular structure to adapt to Hg stress by increasing proline. The other adaptive strategy is via enhancing the elimination of free radicals to mitigate the damage of trace metals through the antioxidant enzyme system (including POD and SOD). At Hg concentration < 20 µM, the increases of POD and SOD can convert more toxic superoxide radicals into harmless ions in plant and remove hydrogen peroxide caused by trace metal stress at the same time^62^. This strategy is to enhance the resistance of *S. caninervis* to Hg by activating the enzymatic system of plant.

## Conclusions

Our results found that Hg stress can change the ultrastructure, SS, Chl, Pro and MDA contents, SOD and POD activities of biocrust moss *S. caninervis.* The growth and physiological functions of *S. caninervis* were significantly inhibited with the increase of Hg concentration. The tolerance threshold of *S. caninervis* to Hg stress was 20 µM. *S. caninervis* can adapt to Hg stress through a series of physiological and ultrastructural changes. Changes in SOD and POD activities, Chl, SS and Pro contents were the main adaptation strategies to combat Hg stress. *S. caninervis* is widely distributed in Gurbantünggüt Desert, and this study indicated that it had a certain tolerance to Hg, which, until now, however, has never been reported previously. Therefore, the findings made in this paper can provide basic data and theoretical support for the environmental impact assessment of trace metals (such as Hg) on vegetation in desert area.
